# Ambulatory Intensive Care for Medically Complex Patients at a Health Care Clinic for Individuals Experiencing Homelessness: The SUMMIT Randomized Clinical Trial

**DOI:** 10.1001/jamanetworkopen.2023.42012

**Published:** 2023-11-10

**Authors:** Brian Chan, Samuel T. Edwards, Priya Srikanth, Matthew Mitchell, Meg Devoe, Christina Nicolaidis, Devan Kansagara, P. Todd Korthuis, Rachel Solotaroff, Somnath Saha

**Affiliations:** 1 Section of Addiction Medicine, Division of General Internal Medicine and Geriatrics, Oregon Health & Science University, Portland; 2 Central City Concern, Portland, Oregon; 3 Center to Improve Veteran Involvement in Care, Veterans Affairs Portland Health Care System, Portland, Oregon; 4 Biostatistics Design Program, Oregon Health & Science University, Portland; 5 School of Social Work, Portland State University, Portland, Oregon; 6 School of Public Health, Oregon Health & Science University–Portland State University, Portland; 7 Division of General Internal Medicine, Johns Hopkins University, Baltimore, Maryland

## Abstract

**Importance:**

Intensive primary care interventions have been promoted to reduce hospitalization rates and improve health outcomes for medically complex patients, but evidence of their efficacy is limited.

**Objective:**

To assess the efficacy of a multidisciplinary ambulatory intensive care unit (A-ICU) intervention on health care utilization and patient-reported outcomes.

**Design, Setting, and Participants:**

The Streamlined Unified Meaningfully Managed Interdisciplinary Team (SUMMIT) randomized clinical trial used a wait-list control design and was conducted at a health care clinic for patients experiencing homelessness in Portland, Oregon. The first patient was enrolled in August 2016, and the last patient was enrolled in November 2019. Included patients had 1 or more hospitalizations in the prior 6 months and 2 or more chronic medical conditions, substance use disorder, or mental illness. Data analysis was performed between March and May 2021.

**Intervention:**

The A-ICU included a team manager, a pharmacist, a nurse, care coordinators, social workers, and physicians. Activities included comprehensive 90-minute intake, transitional care coordination, and flexible appointments, with reduced panel size. Enhanced usual care (EUC), consisting of team-based primary care with access to community health workers and mental health, addiction treatment, and pharmacy services, served as the comparator. Participants who received EUC joined the A-ICU intervention after 6 months.

**Main Outcomes and Measures:**

The main outcome was the difference in rates of hospitalization (primary outcome), emergency department (ED) visits, and primary care physician (PCP) visits per person over 6 months (vs the prior 6 months). Patient-reported outcomes included changes in patient activation, experience, health-related quality of life, and self-rated health at 6 months (vs baseline). We performed an intention-to-treat analysis using a linear mixed-effects model with a random intercept for each patient to examine the association between study group and outcomes.

**Results:**

This study randomized 159 participants (mean [SD] age, 54.9 [9.8] years) to the A-ICU SUMMIT intervention (n = 80) or to EUC (n = 79). The majority of participants were men (102 [65.8%]) and most were White (121 [76.1%]). A total of 64 participants (41.0%) reported having unstable housing at baseline. Six-month hospitalizations decreased in both the A-ICU and EUC groups, with no difference between them (mean [SE], −0.6 [0.5] vs −0.9 [0.5]; difference, 0.3 [95% CI, −1.0 to 1.5]). Emergency department use did not differ between groups (mean [SE], −2.0 [1.0] vs 0.9 [1.0] visits per person; difference, −1.1 [95% CI, −3.7 to 1.6]). Primary care physician visits increased in the A-ICU group (mean [SE], 4.2 [1.6] vs −2.0 [1.6] per person; difference, 6.1 [95% CI, 1.8 to 10.4]). Patients in the A-ICU group reported improved social functioning (mean [SE], 4.7 [2.0] vs −1.1 [2.0]; difference, 5.8 [95% CI, 0.3 to 11.2]) and self-rated health (mean [SE], 0.7 [0.3] vs −0.2 [0.3]; difference, 1.0 [95% CI, 0.1 to 1.8]) compared with patients in the EUC group. No differences in patient activation or experience were observed.

**Conclusions and Relevance:**

The A-ICU intervention did not change hospital or ED utilization at 6 months but increased PCP visits and improved patient well-being. Longer-term studies are needed to evaluate whether these observed improvements lead to eventual changes in acute care utilization.

**Trial Registration:**

ClinicalTrials.gov Identifier: NCT03224858

## Key Points

**Question:**

Can a multidisciplinary ambulatory intensive care unit (A-ICU) with low panel size improve health care utilization and patient-reported outcomes over 6 months for medically and socially complex patients with high rates of homelessness?

**Findings:**

In this randomized clinical trial with 159 participants, there were no differences in hospitalizations or emergency department visits at 6 months between patient groups randomized to an A-ICU intervention or enhanced usual care. However, the A-ICU group had more primary care visits and improved patient-reported well-being.

**Meaning:**

Ambulatory intensive care units can have a positive effect on the health and well-being of complex patients, although longer time horizons may be required to reduce hospitalizations and emergency department visits.

## Teaser

This randomized clinical trial examines the effect of a multidisciplinary ambulatory intensive care unit intervention on health care utilization and patient-reported outcomes among medically and socially complex patients experiencing homelessness.

## Introduction

A small number of patients account for a disproportionately large share of health care utilization and costs in the US.^1^ These patients often face social challenges, including homelessness and poverty, that impose barriers to effectively accessing and using non–acute care services.^2^ Federally qualified health centers (FQHCs) serve many of these patients. Some FQHCs are designated health care programs for individuals experiencing homelessness and receive additional funds to enhance service delivery (eg, on-site, low-cost pharmacy, and mental health referral and treatment).^3^ Yet the psychosocial and structural barriers many patients face in accessing services can limit the effectiveness of these programs in reducing acute care utilization.

Ambulatory intensive care units (A-ICUs) are a form of intensive primary care intervention designed to improve health care utilization and outcomes for medically complex patients.^4^ These A-ICUs are stand-alone primary care teams composed of multidisciplinary staff, with lower patient-to-staff ratios and flexible scheduling to better match patient needs.^5^ Evaluations of A-ICU programs have shown mixed results in reducing high-cost utilization,^6,7,8^ in part due to heterogeneity in patient selection and inclusion of patients potentially less likely to benefit from these programs.^9,10,11,12,13,14,15^ Few studies have examined A-ICUs focused on medically complex patients who have experienced homelessness, a particularly high-risk group.^16^ An evaluation of an A-ICU for homeless veterans in the US showed promise in reducing costs using a nonrandomized design.^17^ Subsequent follow-up studies have shown improvements in patient experience,^18^ satisfaction with care and communication,^11,19^ quality of life, and well-being compared with usual care.^20,21^ Qualitative studies^22^ have also suggested benefits of A-ICUs for both patients and staff.^23^ We sought to build on this evidence by conducting a randomized clinical trial of the Streamlined Unified Meaningfully Managed Interdisciplinary Team (SUMMIT), an A-ICU designed for patients with high acute care utilization seen at an FQHC for individuals experiencing homelessness. We hypothesized that the SUMMIT intervention would reduce hospitalizations and emergency department (ED) visits, increase primary care utilization, and improve patient experience, activation, quality of life, and self-rated health at 6 months compared with enhanced usual care (EUC).

## Methods

### Setting and Trial Design

This randomized clinical trial was developed through a community-academic partnership and was approved by the Oregon Health & Science University Institutional Review Board. Participants provided written informed consent. The SUMMIT intervention and trial design were described previously,^5^ and the trial protocol is provided in Supplement 1. The study followed the Consolidated Standards of Reporting Trials (CONSORT) reporting guideline.

The study took place at Central City Concern, an urban FQHC designated to provide health care for individuals experiencing homelessness.^24^ Every year, Central City Concern serves more than 5000 patients with high rates of poverty and has its own housing, mental health, and addiction treatment services. Despite the availability of these services, many patients have high rates of ED visits and hospitalizations. In 2015, clinic leadership developed the SUMMIT A-ICU to address the needs of their medically complex patients.^4^ The SUMMIT trial was funded through clinic operations as well as from a local payor that included a per-member per-month rate to offset lost visit volume.

The clinic engaged its academic partner to conduct an evaluation of the SUMMIT intervention. Over the course of planning meetings among the research team, clinic staff and leadership, and clinic patient advisory board, we determined that a 6-month wait-list control design would allow all participants to eventually receive the SUMMIT intervention while preserving the evaluative benefits of randomization. Here, we present our 6-month outcomes. Clinic staff implemented the SUMMIT intervention and were unaware of the results until trial completion.

### SUMMIT Intervention

The SUMMIT A-ICU comprised a colocated multidisciplinary team with a reduced panel size (up to 150 patients vs 1000 patients in usual primary care physician [PCP] practice) and flexible scheduling (Box). Staffing consisted of 2 physicians (totaling 1 full-time equivalent) with addiction board certification, a complex care nurse, 2 care coordinators, 2 licensed clinical social workers, a pharmacist, a team manager, and a quality analyst. Team members received training in motivational interviewing, patient goal setting, trauma-informed care, and palliative care through scheduled 2-hour didactics. Core activities included an initial comprehensive patient intake with medical and behavioral team members, patient-driven health goal setting, transitional care protocols when patients experienced hospitalizations, medication management assessment, weekly panel review, and case management to address social determinants of health (eAppendix 1 in Supplement 2).

Box. **Comparison of the SUMMIT A-ICU and EUC Teams**

**SUMMIT A-ICU (1 team, approximately 150 patients)**

- **Care coordinator (2 FTE)**—Serves as the primary point of patient contact. Assists with patient follow-up, acts as a scribe for physician face-to-face visits, and conducts outreach activities with the goal of enhancing rapport building
- **Physician (1 FTE)**—Is a general internist with additional board certification in addiction medicine who provides front-line care to patients, including acute and chronic disease management, advanced care planning, medication management, and coordination of care with specialists
- **Social worker (2 FTE)**—Is embedded in the team as an LCSW who meets with the patients on day 1 to elicit social vulnerabilities and provide counseling plus case management support to patients as needed
- **Complex care nurse (1 FTE)**—Provides medical triage services, facilitates transitional care planning, and assists patients with health education activities and outreach (eg, accompanying patients to specialty appointments)
- **Pharmacist (1 FTE)**—Works with patients and team members to assist with medication reconciliation, transitions of care, and chronic disease (diabetes, heart disease) medication management for patients with the goal of reducing medication treatment burden
- **Team manager (1 FTE)**—Coordinates patient and team schedules, interfaces with clinic operations and administration, conducts outreach, and leads team activities, including organizing trainings and initiating process improvement cycles
- **Quality analyst (0.1 FTE)**—Helps to support quality improvement activities, initiate team PDSA cycles, and develop team reporting dashboards (eg, visit completion, transitions of care process)

**EUC (4 teams, approximately 1500 patients per team)**

- **Practitioner (1 FTE)**—Is a physician, nurse practitioner, physician assistant, or naturopathic practitioner trained in internal medicine or family medicine; some with buprenorphine waiver and/or addiction board certification
- **Medical assistant (1 FTE)**—Rooms patients, takes vital signs, and assists practitioner with scheduling follow-up care appointments and vaccinations
- **Health assistant (1 FTE)**—Has a bachelor’s degree and works in the team room to aid in telephone communication with patients regarding scheduling and/or nonvisit care (eg, communicating results, relaying medication refill requests, or providing advice regarding health issues) and assists the practitioner with obtaining health records and faxing documents (note: each team has a health assistant)
- **Care team manager (1 FTE)**—Is generally an LPN and manages the health assistants, sets schedules, and is available for triage of patient health issues (note: each team has a care team manager)

**Internal patient referrals to**

- **Mental health prescribers (1 FTE)**—On-site psychiatric nurse practitioners who receive referral for medication management
- **Mental health counselors (0.50 FTE)**—On-site LCSWs who are available for referral for short-term counseling (<6 months)
- **Drug and alcohol counseling (1 FTE)**—On-site certified alcohol and drug counselors who perform warm handoffs and referral to treatment services
- **Specialty pharmacy services**—Pharmacy-led chronic disease management for diabetes and hypertension, referred by patient PCP
- **Community health worker (1 FTE)**—Mostly LCSWs trained for brief (<6 months) engagement outside of clinic visits

Abbreviations: A-ICU, ambulatory intensive care unit; EUC, enhanced usual care; FTE, full-time equivalent; LCSW, licensed clinical social worker; LPN, licensed practical nurse; PDSA, plan-do-study-act; SUMMIT, Streamlined Unified Meaningfully Managed Interdisciplinary Team.

### Enhanced Usual Care

The wait-list control group received EUC within Central City Concern. Enhanced usual care consisted of 4 care teams of PCPs, medical assistants, and a care team manager (licensed practical nurse) with access to services including mental health care (staffed by on-site psychiatric nurse practitioners for prescribing and social workers focused on counseling), substance use counseling (brief counseling and referral to treatment completed by certified alcohol and drug counselors), and pharmacy-led interventions (eg, diabetes medication therapy management). In addition, PCPs could refer patients to embedded community health workers who performed short-term (6-month) engagements.^25^ Community health worker activities included motivational interviewing, case management, advocacy, facilitation of multidisciplinary care planning, collaboration with primary care, and individual resource building. Community health workers engaged patients inside and outside the clinic setting.

### Eligibility and Recruitment

Patients were eligible if they spoke English, were aged at least 18 years, had 1 or more hospitalizations in the prior 6 months, and had 2 or more chronic medical conditions (eg, uncontrolled diabetes, congestive heart failure, or end-stage liver disease) or a chronic condition and a substance use disorder or mental illness. Patients were excluded from participation if they were unable to consent due to cognitive impairment (ie, unable to “teach back” consent) or uncontrolled mental illness, had a diagnosis of metastatic cancer or less than 6 months to live, or were unable to participate as a result of aphasia or hearing impairment (eAppendix 2 in Supplement 2). We conducted several meetings with clinic staff to describe SUMMIT, introduce the referral process, and discuss the study design. Primary care physicians submitted referrals for patients to transfer their primary care to SUMMIT. The SUMMIT team members reviewed referrals weekly to confirm suitability for enrollment.

### Enrollment, Randomization, and Follow-Up

Once the patient was accepted to SUMMIT, research staff reviewed the medical record to confirm study eligibility and then contacted the patient to obtain written informed consent and conduct baseline surveys. Research staff then opened an opaque sealed envelope that contained the group assignment generated by a randomization process in R, version 3.3.1 (R Project for Statistical Computing), and they informed the participant. The SUMMIT and EUC teams were then notified of treatment assignment. Six months after randomization, research staff contacted participants, reconfirmed informed consent, and conducted follow-up surveys. At that point, those randomized to EUC were able to join SUMMIT. Patients received a $5 debit card for each survey completed. The first patient was enrolled in August 2016, and the last patient was enrolled in November 2019. During study planning, a power analysis determined that a sample of 200 participants would be able to detect a 30% reduction in hospitalization. Due to resource constraints and the COVID-19 pandemic, recruitment was halted after 159 participants were randomized.

### Measures of Implementation

We assessed implementation^26,27^ by measuring the following: (1) proportion of patients randomized to treatment who had an initial intake appointment with the team; (2) proportion of patients who had at least 3 visits with SUMMIT team members within 6 months; (3) number of visits for each patient, classified by role (eg, physician, nursing, pharmacy, or social work); and (4) length of time spent for each visit, using visit codes within the electronic health record.

### Health Care Utilization Outcomes

The primary outcome was the change in hospitalization rate over 6 months after randomization compared with the 6 months before randomization. Hospitalization rates were calculated by determining the number of hospitalizations per person during both 6-month prerandomization and postrandomization periods. Secondary outcomes included the change in rates of ED, primary care, and behavioral health visits over 6 months.

### Patient-Reported Outcomes

We also assessed the change in patient-reported outcomes at 6 months compared with baseline. We measured patient activation using the 10-item Patient Activation Measure (PAM-10),^28^ which ranges from 0 to 100, with higher scores indicating more activation. Patient experience was measured using the Consumer Assessment of Healthcare Providers and Systems (CAHPS),^29^ with linear mean scoring (0-100) for each of the access, communication, and coordination domains. Higher scores indicate better patient experience. Health-related quality of life was assessed using the 12-item Short Form Survey (SF-12),^30^ generating composites for physical and mental health and for individual domains such as physical functioning, role limitation due to physical health, pain, general health, vitality, role limitation due to emotional problems, social functioning, and mental health. Composite scores are represented as a T-score, with a mean (SD) score of 50 (10); higher scores indicate higher health-related quality of life. We also assessed self-rated health using 1 question from the Edmonton Symptom Assessment Scale (“From a score of 1-10 how do you rate your health?”); higher scores indicated better self-rated health^31^ (eTable 1 in Supplement 2).

### Baseline Survey

The baseline survey included demographics and psychosocial measures to describe our population. Demographic variables included self-reported age, sex, race and ethnicity, household income, and education. Race and ethnicity are reported as American Indian or Alaska Native, Asian, Black or African American, Hispanic or Latino, Native Hawaiian or Other Pacific Islander, White, or other race or ethnicity; these data were collected to provide descriptive information about our study population with regard to generalizability and applicability to other health care settings. Psychosocial variables included perceived social support (using the 7-item ENRICHD [Enhancing Recovery in Coronary Heart Disease Patients] Social Support Instrument),^32^ current living situation,^33^ self-reported health literacy,^34,35^ and food insecurity.^36^ We also screened for depression using the 9-item Patient Health Questionnaire,^37^ for cognitive impairment using the Telephone Interview for Cognitive Status,^38^ and for drug and alcohol use disorders using the Drug Abuse Screening Test and the Alcohol Use Disorder Identification Test.^39^ We also calculated Elixhauser comorbidity scores using patient problem list diagnoses at baseline and stratified them into groups (0, 1-2, 3-4, or ≥5).^40^

### Statistical Analysis

We included all randomized participants in our intention-to-treat analysis. For all outcomes, we report mean within-group changes (from baseline to 6 months) and between-group comparisons of changes (ie, difference) with corresponding 95% CIs. We used linear mixed-effects modeling with a random intercept for each patient to examine the association between study group and outcomes. We chose this model to account for both within-patient correlation and missing data (eg, due to loss to follow-up). Models included an interaction term of time × study group to test for the significance of between-group (difference) comparisons. To quantify the magnitude of influence of outliers on the difference between groups, we conducted sensitivity analyses of outliers by excluding those with large model-based residuals and comparing results from a model with and without outliers. Testing for all analyses was 2 sided at a significance level of *P* < .05 and was conducted using Stata, version 16 (StataCorp). Data analysis was performed between March and May 2021.

## Results

This study randomized 159 participants (mean [SD] age, 54.9 [9.8] years) to the A-ICU SUMMIT intervention (n = 80) or to EUC (n = 79). Patient baseline sociodemographic characteristics were similar in both groups (Table 1). There were 102 men (65.8%) and 53 women (34.2%). In terms of race and ethnicity, 20 participants (12.6%) identified as American Indian or Alaska Native, 3 (1.9%) as Asian, 20 (12.6%) as Black or African American, 5 (3.1%) as Hispanic or Latino, 1 (0.6%) as Native Hawaiian or Other Pacific Islander, 121 (76.1%) as White, and 5 (3.1%) as other race or ethnicity. At baseline, 64 of 156 participants (41.0%) reported having unstable housing. More than half of participants screened positive for at least some drug use (95 of 157 [60.5%]), and 32 of 156 (20.5%) reported an alcohol problem. Most participants (133 of 156 [85.3%]) had incomes of less than $1000 per month, and more than half (96 of 157 [61.1%]) had a high school education or less. The study population averaged 2.5 hospitalizations over the 6 months prior to enrollment. At 6 months, 12 study participants (7.5%) had died (7 of 80 [8.8%] and 5 of 79 [6.3%] in the SUMMIT and EUC groups). Although we had complete follow-up data for our utilization measures, 7 (8.8%) and 6 (7.6%) participants were lost to follow-up in the SUMMIT and EUC groups, respectively (Figure).

**Table 1.** Baseline Characteristics by Intervention Group^a^

Characteristic	All patients (N = 159)	Intervention group	
		SUMMIT (n = 80)	EUC (n = 79)
Age, mean (SD), y	54.9 (9.8)	53.3 (10.3)	56.5 (9.0)
Sex (n = 155)			
Female	53 (34.2)	27 (34.2)	26 (34.2)
Male	102 (65.8)	52 (65.8)	50 (65.8)
Race and ethnicity^b^			
American Indian or Alaska Native	20 (12.6)	10 (12.5)	10 (12.7)
Asian	3 (1.9)	2 (2.5)	1 (1.3)
Black or African American	20 (12.6)	11 (13.8)	9 (11.4)
Hispanic or Latino	5 (3.1)	4 (5.0)	1 (1.3)
Native Hawaiian or Other Pacific Islander	1 (0.6)	1 (1.3)	0
White	121 (76.1)	62 (77.5)	59 (74.7)
Other	5 (3.1)	2 (2.5)	3 (3.8)
Gross household income in past mo, $ (n = 156)			
≤1000	133 (85.3)	68 (86.1)	65 (84.4)
>1000	23 (15.1)	12 (16.0)	11 (14.3)
High school education or less (n = 157)	96 (61.1)	47 (59.5)	49 (62.8)
Social support (ENRICHD score), mean (SD) (n = 157)	18.9 (6.7)	19.8 (6.3)	17.8 (7.0)
Self-rated health, mean (SD) (n = 158)	5.3 (2.3)	5.3 (2.2)	5.3 (2.4)
HRQOL SF-12 aggregate composite, mean (SD) (n = 151)			
Physical health	27.2 (9.2)	25.9 (8.9)	28.6 (9.3)
Mental health	40.1 (13.0)	40.3 (12.5)	39.8 (13.6)
Patient activation (PAM-10 score), mean (SD) (n = 158)	55.6 (12.0)	56.6 (12.6)	54.6 (11.3)
Cognitive impairment (TICS score <20^38^) (n = 155)	63 (40.6)	30 (38.5)	33 (42.9)
Drug abuse screening (DAST-10 score^39^) (n = 157)			
No problems reported (0)	62 (39.5)	28 (35.4)	34 (43.6)
Low or moderate level (1-5)	56 (35.7)	31 (39.2)	25 (32.1)
Substantial or severe level (>5)	39 (24.8)	20 (25.3)	19 (24.4)
Current alcohol problem (AUDIT-10 score >7) (n = 156)	32 (20.5)	16 (20.5)	16 (20.5)
Current residence (n = 156)			
Sleeping outside or at a place not meant for habitation	21 (13.4)	7 (9.0)	14 (17.9)
Shelter	15 (9.6)	11 (14.1)	4 (5.1)
Transitional housing	28 (18.0)	11 (14.1)	17 (21.9)
Permanent housing	62 (39.8)	31 (39.8)	31 (39.8)
With friends or family	8 (5.1)	5 (6.4)	3 (3.8)
Nursing facility or assisted living	22 (14.1)	13 (16.7)	9 (11.5)
Housing stability (n = 156)^c^			
Unstable	64 (41.0)	29 (37.2)	35 (44.8)
Stable	92 (59.0)	49 (62.8)	43 (55.2)
Presence of depression (PHQ-9 score >9) (n = 156)	84 (53.8)	39 (49.4)	45 (58.4)
Elixhauser comorbidity score (n = 155)			
0	59 (38.1)	29 (37.2)	30 (39.0)
1-2	4 (2.6)	3 (3.8)	1 (1.3)
3-4	38 (24.5)	19 (24.4)	19 (24.7)
≥5	54 (34.8)	27 (34.6)	27 (35.1)
Inadequate health literacy (n = 156)^d^	79 (50.6)	37 (46.8)	42 (54.6)
Food insecurity (n = 154 and 155)^e^	101 (65.6)	51 (67.1)	50 (64.1)

Abbreviations: AUDIT-10, 10-item Alcohol Use Disorders Identification Test; DAST-10, 10-item Drug Abuse Screening Test; ENRICHD, Enhancing Recovery in Coronary Heart Disease Patients Social Support Instrument; EUC, enhanced usual care; HRQOL SF-12, health-related quality of life 12-item Short Form Survey; PAM-10, 10-item Patient Activation Measure; PHQ-9, 9-item Patient Health Questionnaire; SUMMIT, Streamlined Unified Meaningfully Managed Interdisciplinary Team; TICS, Telephone Interview Cognitive Status.

^a^ Unless indicated otherwise, values are presented as No. (%) of patients.

^b^ Percentages may not total 100% because of rounding and multiple race and ethnicity categories checked.

^c^ Unstable housing was defined as sleeping outside or in a place not meant for habitation, shelter, or transitional housing; stable housing was defined as permanent housing, staying with friends or family, or living in a nursing facility or assisted living.

^d^ Inadequate health literacy was defined as the percentage responding “not at all/little bit/moderately” confident in filling out forms by themselves.^35^

^e^ Food insecurity was defined as an affirmative response on either question 1 (“In the last 12 months, did you ever eat less than you felt you should because there wasn’t enough money for food?”) or question 2 (In the last 12 months, were you ever hungry but didn’t eat because there wasn’t enough money for food?”).^36^

**Figure.**
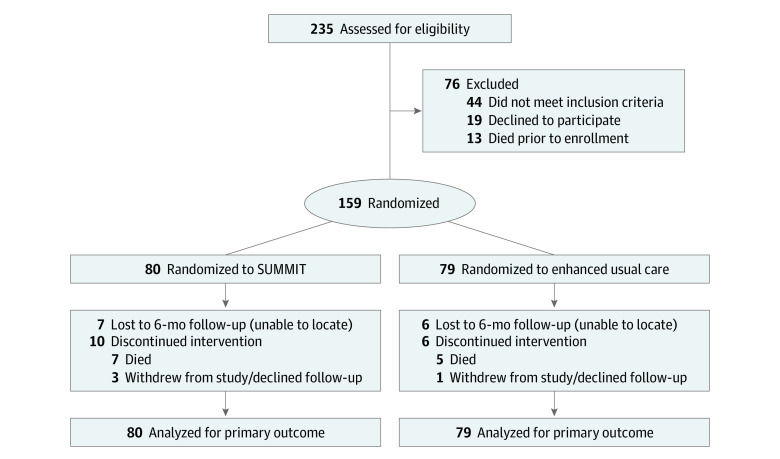
Screening, Randomization, and Analysis Flow Diagram

### Implementation Measures

Among participants randomized to the SUMMIT intervention, all completed the initial intake appointment. At 6 months, 70 patients (87.5%) had 3 or more visits with the team. On average, each SUMMIT patient had 36 appointments with team members and averaged 40 minutes per visit. This included 69 patients (86.3%) with 1 or more mental health visits and 67 patients (83.8%) with 2 or more visits with a physician. More than half of visits were with physicians (429 visits [28.2%]) or social workers (374 visits [24.6%]).

### Health Care Utilization Outcomes

The SUMMIT and EUC groups experienced similar declines in mean (SE) 6-month hospitalization rates (SUMMIT vs EUC within-group change, −0.6 [0.5] vs −0.9 [0.5] hospitalizations per person; difference, 0.3 [95% CI, −1.0 to 1.5]). Both groups also had similar reductions in mean (SE) 6-month ED visit rates (−2.0 [1.0] vs −0.9 [1.0]; difference, −1.1 [95% CI, −3.7 to 1.6]). The SUMMIT participants experienced significantly greater increases in mean (SE) primary care visits (4.2 [1.6] vs −2.0 [1.6] visits per person; difference, 6.1 [95% CI, 1.8 to 10.4]) and behavioral health visits (4.7 [1.1] vs −1.1 [1.1] visits per person; difference, 5.8 [95% CI, 2.8 to 8.8]) relative to EUC participants (Table 2).

**Table 2.** Utilization Outcomes at 6 Months From a Linear Mixed-Effects Model

Outcome	Intervention group, mean (SE)^a^						Between-group difference (95% CI)	*P* value
	SUMMIT (n = 80)			EUC (n = 79)				
	Baseline	6 mo	Within-group change	Baseline	6 mo	Within-group change		
Primary								
Hospitalization rate	2.7 (0.4)	2.04 (0.4)	−0.6 (0.5)	2.8 (0.4)	1.9 (0.4)	−0.9 (0.5)	0.3 (−1.0 to 1.5)	.68
Secondary								
ED visit rate	5.5 (0.7)	3.5 (0.7)	−2.0 (1.0)	3.9 (0.7)	2.9 (0.7)	−0.9 (1.0)	−1.1 (−3.7 to 1.6)	.43
Primary care visit	7.8 (1.2)	12.0 (1.2)	4.2 (1.6)^b^	7.4 (1.2)	5.4 (1.2)	−2.0 (1.6)	6.1 (1.8 to 10.4)	.005
Behavioral health visit	3.3 (1.6)	7.9 (1.6)	4.7 (1.1)^b^	6.8 (1.6)	5.6 (1.6)	−1.1 (1.1)	5.8 (2.8 to 8.8)	<.001

Abbreviations: ED, emergency department; EUC, enhanced usual care; SUMMIT, Streamlined Unified Meaningfully Managed Interdisciplinary Team.

^a^ Random intercept for each patient.

^b^ *P* < .01.

### Patient-Reported Outcomes

Both groups experienced increases in mean (SE) PAM-10 scores at 6 months (SUMMIT vs EUC within-group change, 3.5 [1.6] vs 2.3 [1.6]; difference, 1.2 [95% CI, −3.3 to 5.7]). Among the 3 CAHPS domains, SUMMIT participants reported increases in access (mean [SE], 5.7 [3.9] vs 4.0 [3.9]; difference, 1.7 [95% CI, −9.1 to 12.5]), communication (mean [SE], 5.2 [3.5] vs −3.7 [3.5]; difference, 9.0 [95% CI, −0.7 to 18.7]), and coordination (mean [SE], 9.8 [4.4] vs 0.5 [4.4]; difference, 9.2 [95% CI, −3.1 to 21.6]) relative to EUC participants, although these differences between groups were not significant. The SUMMIT participants also experienced nonsignificant increases in mean (SE) SF-12 physical health composite scores (2.5 [1.1] vs 0.7 [1.1]; difference, 1.8 [95% CI, −1.3 to 4.9]) and SF-12 mental health composite scores (2.2 [1.7] vs 0.1 [1.7]; difference, 2.2 [95% CI, −2.5 to 6.8]) relative to EUC participants. The SUMMIT participants compared with EUC participants had significantly greater increases in the SF-12 social functioning subdomain (mean [SE], 4.7 [2.0] vs −1.1 [2.0]; difference, 5.8 [95% CI, 0.3 to 11.2]) and in self-rated health (mean [SE], 0.7 [0.3] vs −0.2 [0.3]; difference, 1.0 [95% CI, 0.1 to 1.8]) (Table 3). Excluding outliers did not change our conclusions (eTables 2 and 3 in Supplement 2).

**Table 3.** Patient-Reported Outcomes at 6 Months From a Linear Mixed-Effects Model

Measure	Intervention group, mean (SE)^a^						Between-group difference (95% CI)	*P* value
	SUMMIT (n = 80)			EUC (n = 79)				
	Baseline	6 mo	Within-group change	Baseline	6 mo	Within-group change		
PAM-10^28^	56.6 (1.3)	60.1 (1.5)	3.5 (1.6)^b^	54.6 (1.3)	57.0 (1.5)	2.3 (1.6)	1.2 (−3.3 to 5.7)	.61
CAHPS domain^29^								
Access	68.9 (2.9)	74.5 (3.3)	5.7 (3.9)	68.9 (3.0)	72.9 (3.3)	4.0 (3.9)	1.7 (−9.1 to 12.5)	.76
Communication	76.6 (2.7)	81.8 (3.1)	5.2 (3.5)	84.8 (2.7)	81.0 (3.0)	−3.7 (3.5)	9.0 (−0.7 to 18.7)	.07
Coordination	64.3 (3.3)	74.1 (3.7)	9.8 (4.4)^c^	73.7 (3.3)	74.2 (3.7)	0.5 (4.4)	9.2 (−3.1 to 21.6)	.14
HRQOL SF-12^30^								
Physical health composite	25.8 (1.0)	28.3 (1.2)	2.5 (1.1)^b^	28.4 (1.1)	29.0 (1.1)	0.7 (1.1)	1.8 (−1.3 to 4.9)	.26
Mental health composite	40.3 (1.5)	42.5 (1.7)	2.2 (1.7)	40.0 (1.5)	40.0 (1.6)	0.1 (1.7)	2.2 (−2.5 to 6.8)	.36
Physical functioning	29.2 (1.1)	29.9 (1.2)	0.7 (1.3)	29.4 (1.1)	29.9 (1.2)	0.5 (1.3)	0.3 (−3.5 to 4.0)	.90
Role limitation due to physical health	29.4 (1.0)	31.6 (1.2)	2.2 (1.3)	30.9 (1.0)	31.8 (1.1)	0.9 (1.3)	1.3 (−2.4 to 5.0)	.50
Pain	27.0 (1.5)	29.0 (1.6)	2.0 (1.6)	30.6 (1.5)	29.7 (1.6)	−0.9 (1.6)	2.9 (−1.6 to 7.4)	.20
General health	25.9 (1.1)	30.7 (1.2)	4.8 (1.4)^c^	27.4 (1.1)	29.9 (1.2)	2.4 (1.4)	2.4 (−1.5 to 6.3)	.24
Vitality	42.2 (1.3)	41.5 (1.5)	−0.7 (1.5)	39.3 (1.4)	40.4 (1.5)	1.1 (1.5)	−1.9 (6.1 to 2.4)	.39
Role limitation due to emotional problem	34.1 (1.5)	36.5 (1.7)	2.3 (2.1)	33.6 (1.5)	34.3 (1.7)	0.7 (2.0)	1.6 (−4.1 to 7.3)	.58
Social functioning	29.7 (1.5)	34.4 (1.7)	4.7 (2.0)^b^	34.1 (1.5)	33.1 (1.7)	−1.1 (2.0)	5.8 (0.3 to 11.2)	.04
Mental health	38.6 (1.4)	40.9 (1.5)	2.3 (1.5)	38.9 (1.4)	38.7 (1.5)	−0.2 (1.5)	2.5 (−1.7 to 6.6)	.24
Self-rated health^31^	5.3 (0.3)	6.0 (0.3)	0.7 (0.3)^b^	5.2 (0.3)	5.0 (0.3)	−0.2 (0.3)	1.0 (0.1 to 1.8)	.03

Abbreviations: CAHPS, Consumer Assessment of Healthcare Providers and Systems; EUC, enhanced usual care; HRQOL SF-12, health-related quality of life 12-item Short Form Survey; PAM-10, 10-item Patient Activation Measure; SF-12, 12-item Short Form Survey; SUMMIT, Streamlined Unified Meaningfully Managed Interdisciplinary Team.

^a^ Random intercept for each patient.

^b^ *P* < .05.

^c^ *P* < .01.

## Discussion

In this community-partnered randomized clinical trial of an A-ICU intervention for medically and socially complex patients experiencing homelessness, we found no differences in hospitalizations at 6 months between those receiving A-ICU care and those receiving EUC. However, the intervention increased outpatient visits and improved patient-reported social functioning and self-rated health. These findings suggest that A-ICU and other intensive primary care interventions may have positive effects on the care of high-need, high-cost patients but may not achieve the goal of reduced acute care utilization, at least in the short term.

To our knowledge, this is the first randomized clinical trial to assess the effect of an A-ICU model for patients at a FQHC with high rates of poverty and acute care utilization. Our intervention and population were similar to those in the Veterans Affairs (VA) Homeless Patient Aligned Care Team program. Data from that program showed reduced costs^41^ and improved patient experience^42^ but were based on nonrandomized evaluations. Other intensive primary care interventions, including other VA intensive management programs,^11,19^ the VA Home Based Primary Care program,^43^ and the US Centers for Medicare & Medicaid Services Program of All-Inclusive Care for the Elderly,^44^ have targeted populations different from ours and have shown mixed results in terms of utilization and cost reductions. The Camden Coalition study was, like ours, a randomized trial of intensive outpatient management among low-income patients, although it used a different model of care (hospital-based care-transitions program),^12^ and showed no differences in 6-month readmission rates.

Several factors may explain why 6-month hospitalization rates did not improve. First, our EUC comparator was a high-functioning patient-centered medical home with high levels of patient access, staff members with experience in culturally appropriate care, and links to CHWs^45^ and social services referral pathways. Second, regression to the mean, a common phenomenon when selecting patients based on high rates of acute care utilization, was evident in our study; both the intervention and control groups experienced reductions in hospitalization. Ideally, interventions should target patients destined to have persistently high utilization rates, but predicting future utilization patterns is challenging.^12,13,46,47^ Finally, for medically complex patients with limited access to care, hospitalizations might be necessary to address “pent-up” patient needs in the short term; qualitative data from interviews with our A-ICU team indicated that this was an issue for some enrollees.^23^

Our results and those of other studies suggest that 6 months may be too short a period to see changes in hospitalization rates. For our population of patients living in high poverty and experiencing high stigmatization with years of negative interactions with the health care system, effective use of primary and acute care services may require years and involve building rapport with clinicians, trust in health care institutions, and skills in navigating complex health care systems. In qualitative interviews, SUMMIT team members noted that our current health care system is not designed to meet the needs of this patient population.^23^

The SUMMIT intervention led to more outpatient primary care and behavioral health visits, higher social functioning, and better self-reported health. These findings are consistent with those of prior studies of intensive primary care in the VA, which showed similar increases in outpatient visits and patient satisfaction.^11,18,19^ These improvements demonstrate important benefits that intensive primary care programs may have in the short term, which may be an intermediate step on the path to longer-term changes in acute care utilization. Interviews we conducted with patients in our study suggest that the connection they developed to the SUMMIT team through increased visits, the structure of the visits (longer appointments with multiple team members), and team activities (huddles, weekly rounds, outreach) that fostered shared knowledge of the patient story were all factors that patients recognized and may have led to improved well-being and engagement.^48^

Our results must also be interpreted in the context of the social and health-related challenges faced by SUMMIT patients. Although SUMMIT was designed to address social determinants underlying the poor health of its patients, A-ICU interventions like SUMMIT are effectively bandages for deep wounds caused by the social deprivation faced by the patients they serve. The effectiveness of intensive primary care interventions may be limited without larger policy and community-level systems changes necessary to address the living and working conditions that lead to poverty, poor health outcomes, and health inequity.^6^

### Limitations

This study has some limitations. First, we were unable to reach our recruitment target. Second, although attrition was balanced across groups and was relatively low for this difficult-to-reach population, it reduced the statistical power of our patient-reported outcomes. However, the lack of change in the primary outcome between groups suggests that we would not have observed a statistically significant improvement in hospitalization rates even if we had reached our sample size target. Third, we had a relatively high number of deaths during the 6-month period, indicating the frailty of our population. Finally, we relied on PCPs to refer patients for the trial; it is unclear how nonreferred complex patients may have fared. Although the intended target was patients with medical complexity as a driver of their hospitalizations, it is difficult to disentangle behavioral health and substance use drivers of hospitalization, which may require different interventions.

## Conclusions

In this randomized clinical trial including medically and socially complex patients at an urban FQHC with high rates of acute care utilization, our A-ICU intervention did not reduce hospitalizations or ED visits at 6 months but increased primary care visits and improved patient-reported social functioning and self-reported health status. These programs provide important benefits beyond utilization reductions. Short-term utilization reduction may not be achievable with intensive primary care programs alone, but whether their short-term benefits lead to longer-term effects on utilization merits further study.

## Notes

Supplement 1.
Trial Protocol

Supplement 2. **eAppendix 1.** Core Activities of the Streamlined Unified Meaningfully Managed Interdisciplinary Team (SUMMIT) Ambulatory Intensive Care Unit (A-ICU) **eAppendix 2.** Study Enrollment Criteria **eTable 1.** Outcomes and Assessment Instruments **eTable 2.** Outlier Sensitivity Analysis for Administrative and Patient-Reported Outcomes at 6 Months From a Linear Mixed-Effects Model **eTable 3.** Missing Data Sensitivity Analysis of Baseline Characteristics of Those Missing Patient-Reported Outcomes at 6 Months
eReferences

Supplement 3.
Data Sharing Statement
