# Supplementary material for: Ambulatory Intensive Care for Medically Complex Patients at a Health Care Clinic for Individuals Experiencing Homelessness: The SUMMIT Randomized Clinical Trial
Source: JAMA Netw Open. 2023 Nov 10;6(11):e2342012. doi: 10.1001/jamanetworkopen.2023.42012 (PMC10638646; doi:10.1001/jamanetworkopen.2023.42012)
Supplement: Supplement 1. — Trial Protocol [file jamanetwopen-e2342012-s001.pdf]

Minimal Risk Protocol

1) Protocol Title

VA/OHSU (J): Does a clinic based complex care coordination intervention improve patient quality outcomes in an underserved clinic population? The Streamlined, Unified, Meaningfully Managed Interdisciplinary Team (SUMMIT) Ambulatory ICU Study

2) Objectives

This is a prospective cohort, wait-list control design study comparing a complex care management (CCM) intervention versus usual care on Patient Activation Measure (PAM) scores, healthcare utilization measures, and health outcomes in a single clinic population served at Federally Qualified Health Center (FQHC) in Portland, Oregon.

Primary Research Question:

Does a team-based complex care multi-disciplinary team focused on a high risk population in an underserved community FQHC improve healthcare utilization outcomes, including inpatient hospitalization costs, number of hospitalizations for ambulatory care sensitive conditions (ACSC) per patient, early readmission, number of ED, number of primary care visits?

Secondary Research Questions:

Secondary research questions include: In a high risk patient population at a single FQHC, does a team-based complex care multi-disciplinary team improve (1) costs of care over 6-12 months compared to the prior 6-12 months, (2) patient experience at 6 months compared to baseline measurement, (3) patient activation at 6 months compared to baseline measurement, (4) functional status at 6 months compared to baseline measurement, and (5) patient reported chaos at 6 and 12 months compared to baseline measurement.

3) Background

There is increased interest in addressing the needs of "high utilizers," the small proportion of patients who account for a large percentage of health care expenditures.[1] These patients often have multiple medical comorbidities that influence clinical practice patterns and outcomes.[2] Patients with multiple medical conditions are more likely to have functional impairments[3] and experience adverse drug events.[4] The number of specialty physicians involved in management of multiple medical conditions also increase risk of fragmentation of care coordination,[5] and poor transitions of care from hospital to home.[6-8] In addition, social factors, such as low socioeconomic status (SES), homelessness, and substance use disorders are risk factors for being hospitalized for ambulatory care-sensitive conditions, unnecessary ED visits, readmissions, and multiple hospitalizations, yet less likely to use primary care than their higher SES counterparts.[9]

The dissemination of Accountable Care Organizations (ACO) have led to provider groups assuming more financial risk for the quality of care and costs of high need and high cost patients. Efforts to discover and implement complex care management teams (CCM) are underway in effort to improve outcomes and reduce costs by addressing the needs of this population.[10] A recent comparison of the operational approaches of 18 CCM program provided guidance on "best practices."[11] However, there is still limited evidence to suggest which interventions CCM

teams should adopt in order to address the needs of medically and socially vulnerable patient populations. Furthermore, it is unclear whether specific subsets of patients would benefit more from CCM teams.

An AHRQ systematic review of 17 comparative studies of PCMH models of care concluded PMCH models had no effect on hospital admissions, but did reduce emergency room visits by 19% and improved staff and patient experience.[12] This has led to development of high-intensity primary care programs, also called ambulatory intensive caring units, which include elements of PCMH targeted toward the sickest, highest cost patients.[13] A second systematic review of these programs analyzed 20 studies of home-based and free-standing intensive primary care programs.[14] These studies were assessed as moderate evidence, with inconsistent findings on whether these models of care reduced hospital admission, hospital days, and ED department use outcomes. None of the programs evaluated reduced mortality. Current gaps in the literature include lack of quality studies that include comparison of control populations, and study of patient populations with high substance use, mental health illness, and homelessness, risk factors for high utilization of care services. An RCT trial of a complex care management program with longitudinal care coordination, motivational interviewing, and addictions and social work assistance targeted toward alcohol and drug dependent participants showed no effect of self reported abstinence,[15] but the intervention did not target improved medical comorbidity.

4) Study Design

<u>Intervention Design:</u>

<u>Participants receiving usual care at Old Town Clinic (OTC) have access to the following services:</u>

1 Continuation of care with existing primary care provider at OTC who provides either 30 minute (for new patients establishing at Old Town Clinic), or 15 minute follow up appointments for clinical care. Usual care providers on average have a panel of 1000 patients for 1 FTE. Providers are supported by a medical assistant (MA) with a ratio of 1 MA for 2 Providers, a Care Team Manager who is a licensed practical nurse (LPN), and 1 health assistant (BA) who assists in clerical duties

2 In addition to routine appointment scheduling, participants have access to “same day appointment” scheduling, requiring participants to call 24 hours in advance of the appointment to be seen.

3 Participants have access to internal referrals to specialty care services provided at Old Town Clinic including, wellness groups, acupuncture, alcohol and substance abuse counseling services (A&D), tobacco cessation counselling, pharmacy management consultations, and mental health counseling services provided by (psychiatric mental health nurse practitioner) PMHNP and (licensed clinical social worker) LCSW trained staff.

4 Participants have access to external referrals to specialty care services through referrals made by the primary care provider and facilitated by a referrals coordinator assigned to the providers.

The SUMMIT team differs from usual care at OTC in terms of team staffing, panel size, training, and opportunity for collaboration. SUMMIT intervention participants will receive: (see figure 1)

1. **Transfer to a stand-alone, co-located team:** Re-assignment of their primary care provider to the SUMMIT team, a co-located team of MD, MA, behavioral health outreach worker (BHOW) who are qualified mental health providers (QMHP), a team pharmacist, led by a team manager (BA) and quality and data specialist (MA). The patient to provider ratio is significantly reduced (200 patients: 1 MD FTE). All members will share a team room in the OTC building to facilitate interdisciplinary collaboration and communication. Team members participate in daily huddles to discuss patients on the day's schedule, conduct panel management activities, as well as team building mindfulness activities.

2. **Intensive intake process:** The new patient intake consists of a 30-minute visit with the care coordinator, who update patients' histories, needs and goals, and up to 60 minutes with the physician. The long intake visit provides the opportunity for trust building and to ensure that the team has a full picture of the patients' care goals. Team members will use rapport building, motivational interviewing, and relationship building to elicit patient values, with the aim to develop a comprehensive care plan.

3. **On-demand availability:** The SUMMIT team uses open-access scheduling with longer, more flexible appointments to accommodate patients' needs on short notice. Additionally, the physicians are available through a "warm-line" paging service 24-hours a day. Flexibility and ability to coordinate transitions of care after hospitalization episodes, or conduct outreach visits to foster engagement and conduct wellness checks.

4. **Semi-autonomous:** SUMMIT strives to keep as much care as possible centralized within the team. The team consults with outside specialist "champions" to reduce the need for outside specialist appointments. Physicians have additional training in treating substance use disorders, psychopharmacology and palliative care, in order to reduce internal referral. Behavioral health counseling is available within the team through the BHOW. This centralization of care and services aims to build trusting relationships to educate patients on their illnesses and promote self-management skills.

5. **Navigation of social services:** Behavioral health outreach workers provide case management to patients who need referrals to social services. Benefits support, short-term rental assistance, and flexible funds are available.

6. **Provider and team wellness:** The SUMMIT team incorporates mindfulness activities into daily huddles, and the team manager checks in with team members regularly to ensure they have the resources and support needed. Small patient panels and increased time for both medical visits and care coordination enhance staff satisfaction.

<u>Figure 1: SUMMIT intervention logic model</u>

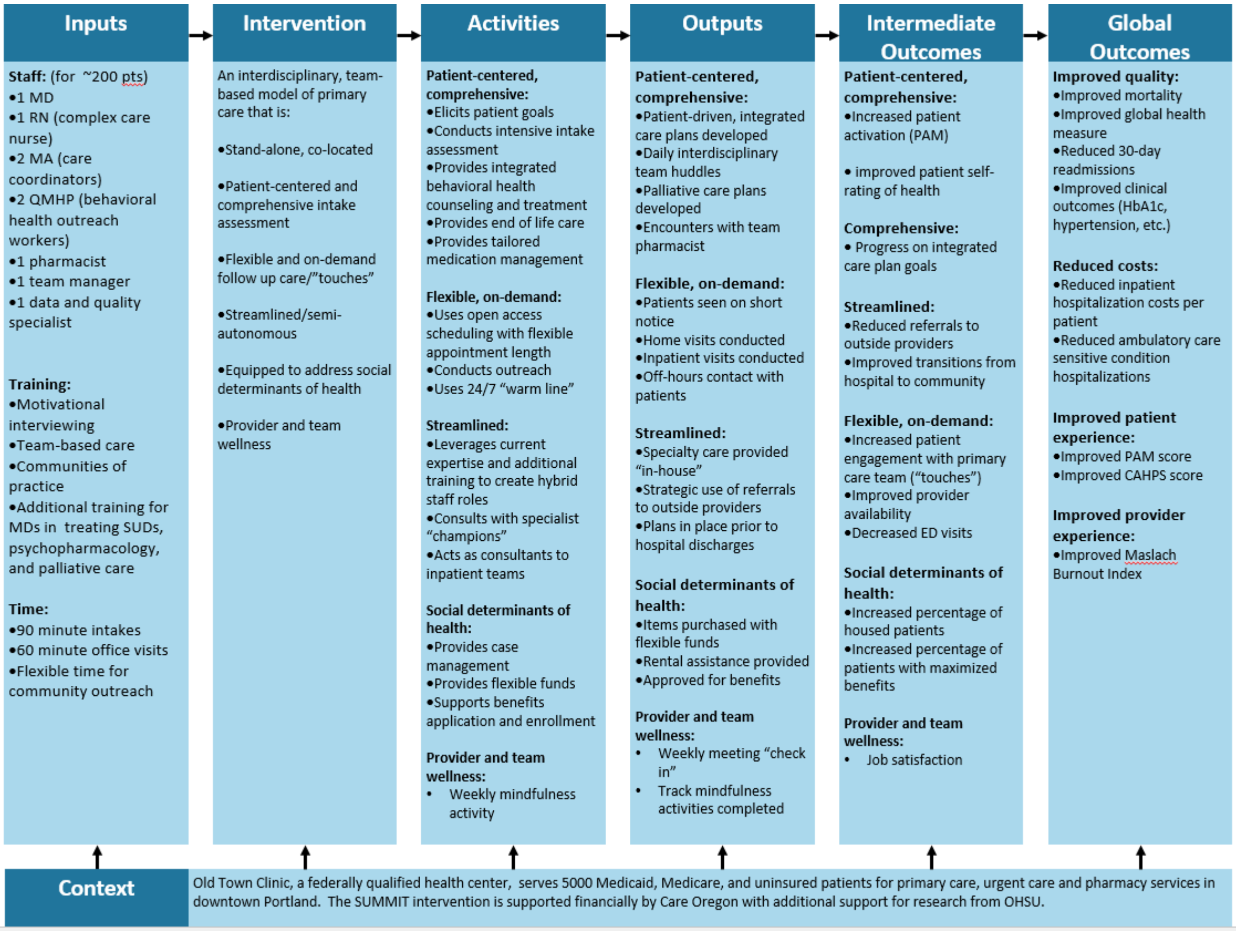

Study Design and Evaluation Plan:

This will be a controlled quasi-experimental evaluation trial with a randomized wait-list control design, which has been described in prior implementation science literature.[16,17] This design is notable for staggered introduction of the intervention over time to all target participants, opportunity for cross-over of participants from control to intervention arm, and regular data collection points (see figure 2). Compared to a parallel group randomized clinical trial, a wait-list control design was more acceptable to Old Town Clinic leadership in that it was felt to be unethical for some of the target population not to receive the intervention at all. Second, this design is practical as the intervention staffing and recruitment will scale up over time. Conducting several recruitment waves will allow for a dynamic cohort of participants to participate in the intervention, and allows the analysis to take into account variations over time.

Figure 1. Quasi-experimental evaluation trial design of SUMMIT intervention, a complex care coordination intervention

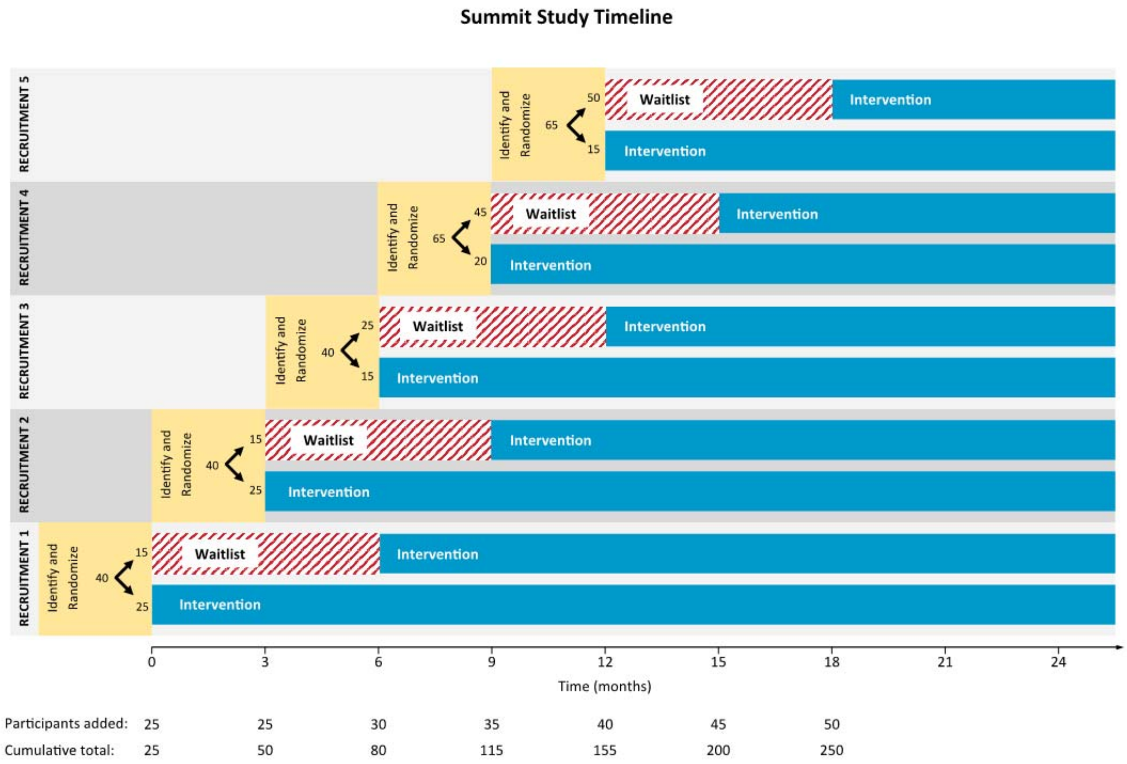

Fig 2. Proposed Study Timeline

We also plan to supplement our quantitative evaluation of the intervention with a qualitative component to obtain additional insights from both participant and provider perspectives. Ethnographic data collection methods will be used to gain an in-depth understanding of how the different SUMMIT components function to reduce Ambulatory Care Sensitive Conditions (ACSC) related hospitalizations through detailed examination and documentation of practice structure, work flow processes, and patient perspective of service delivery. Methods for ethnographic data collection are divided as follows: purposive sampling of participants, semi-structured interviews with SUMMIT and usual primary care staff, participant observation of SUMMIT team activities, and questionnaires with participants enrolled in SUMMIT intervention and with participants in "usual care" who are awaiting the intervention. A purposive sampling strategy will be used to identify prospective research participants. Semi-structured interviews will be used to collect in-depth, qualitative data from SUMMIT staff members regarding the structure and work. Semi-structured interviews will be audio recorded and transcribed verbatim into electronic documents for subsequent data analysis. A trained participant-observer will take structured field notes at SUMMIT staff meetings. Using data garnered from interviews with SUMMIT staff and field notes, domains and associated questions relevant to management of medically and social complex patients will be identified and incorporated into an open-ended questionnaire designed to elicit patient perspectives of care delivery in the context of A-ICU model of care and regular primary care. All textual data will be analyzed according to the principles of constructivist grounded theory, a set of systematic techniques for the inductive identification of patterns in human experience. A member of the research team will use the software program ATLAS.ti for data management and analysis. The goal of this phase of data collection and analysis will be to construct and confirm a conceptual model of an A-ICU intervention that illustrates the linkages between different themes emergent in interview, observational, and questionnaire data.

5) Study Population

a) Number of Subjects

Approximately 400 patients at Old Town Clinic will receive the intervention, about 10% of the 4,000+ active patient population. We plan to enroll 300 patient participants in the study. In addition we plan to enroll 80 provider/staff participants to participate in the study, for a total of 380 participants.

b) Inclusion and Exclusion Criteria

Eligibility screening: Potential study subjects will be identified through a referral from a medical provider at OTC using guidelines developed from a prior needs analysis that considered the following factors:

Medical burden – One or more of the following medical conditions: congestive heart failure, uncontrolled diabetes, end stage liver disease, chronic kidney disease (stage III or higher), chronic obstructive pulmonary disease (group C or D), chronic or severe soft tissue infections or ulcers, osteomyelitis, or failure to thrive

Behavioral health – One or more of the following behavioral health conditions: psychotic disorder, mood disorder, post-traumatic stress disorder, or active substance use disorder

Utilization – One or more of the following utilization patterns: high primary care utilization (>10 visits in previous 12 months), low primary care utilization (<5 visits in previous 12 months), frequent missed appointments (cancel or no-show for >5 primary care or specialty appointments in previous 12 months), or recent hospitalizations (>1 medical hospital admission in previous 6 months)

Patients who did not meet the specific enrollment criteria but required the intensive SUMMIT intervention were considered on a case-by-case basis.

Exclusion criteria: Non-English speaking patients will be excluded from the study. Patients on hospice,; inability to consent (as demonstrated by teach back of the consent process); diagnosis of metastatic brain cancer; inability to participate in follow up phone due to aphasia, severe hearing impairment, or lack of access to telephone. In addition, those participants whose degree of substance abuse disorder or behavioral health diagnosis warrants enrollment in another OTC program will be excluded from participation in the SUMMIT A-ICU.

In the event of a screen failure, collected data will be retained for 6 years after the study cut-off period (expected to be end of FY2018) before being destroyed, as per Records Control Schedule policy.

c) Vulnerable Populations

This study will exclude members of vulnerable populations, including children, pregnant women, neonates, decisionally impaired adults, and prisoners. As above, non-English speaking patients will be excluded from the study.

d) Setting

Oregon Health & Science University (OHSU) is a non-profit public corporation and academic medical center in Portland, Oregon.

The Old Town Clinic (OTC) is an integrated medical and behavioral healthcare clinic for homeless individuals or those with very low incomes. OTC is one of 30 primary care practices recently selected as an "exemplar practice" by the Robert Wood Johnson Foundation's LEAP Project. Over 3,500 individuals received services last year. In 2006, OHSU and OTC initiated a partnership with OHSU supporting a medical directorship, with the goal of working together to identify grant and research opportunities in the areas of healthcare disparities, integrated care models and improved access for the underserved.

e) Recruitment Methods

Recruitment will occur in several waves from planned study initiation (spring 2016), each wave will consist of identification of 40-60 potential SUMMIT candidates that meet inclusion criteria. (see figure 1) Usual care providers at OTC will be able to initiate a referral of potential SUMMIT patients by completion of a referral form (see appendix). Referrals will be reviewed monthly by SUMMIT team staff to identify participants appropriate for the intervention based on pre-defined criteria of medical and social complexity. Upon acceptance of the SUMMIT referral, the trained study assistant will review the candidate's appropriateness for study participation with both the participants existing primary care team and SUMMIT care team.

f) Consent Process

If the participants meet inclusion criteria, staff will review the medical chart to review exclusion criteria. If no exclusion criteria are met, a trained study staff /research assistant will contact the participant, explain the study and obtain consent. Trained study staff/research assistant will meet with participants at OTC to explain the study and to obtain consent. A written consent form will be presented to the participants and discussed along with a trained study assistant; alternatively, an online consent form will be presented to the participant using REDCap database software on a computer tablet. The patient must be able to demonstrate understanding via teach-back. Patients will be informed that they may receive the intervention regardless of whether they participate in the study. We are requesting a waiver of documentation of consent for participants whose consent is obtained using REDCap.

Adults Unable to Consent/Decisionally Impaired

In order to consent, the patient must be able to demonstrate understanding via teach-back to a trained study staff/research assistant. There is an expectation that a proportion of the target population may have intermittent decisional impairment (ie. may be acutely intoxicated). In these situations, if the person is unable to demonstrate understanding via teach-back the patient will be excluded from participation in the study. If at a later date, the potential participant meets inclusion criteria and is able to consent, he or she may be approached to consent at that time.

6) Procedures

If deemed appropriate for participation in the study, a trained study assistant will meet the patient, explain the study, answer questions, and obtain consent for participation. The patient must be able to demonstrate understanding via teach-back. Once consent is obtained, the trained study assistant will go

through the baseline survey with participant using a laptop computer (see attached baseline survey instrument). Following completion of the baseline survey, the participant will be randomized by the Redcap randomization module to immediate entrance into the SUMMIT intervention or placement on the 6 month waitlist. Upon obtaining the group assignment, the participant and clinical staff will be made aware of the intervention assignment.

If the participant is randomized to wait-list control, the participant will resume usual care for 6 months. After 6 months have elapsed, the trained study assistant will contact the participant to repeat the baseline survey, and the participant will then undergo formal intake procedures by the SUMMIT team.

If the participant is randomized to the intervention, the participant will undergo formal intake procedures by the SUMMIT team. Once enrolled in the SUMMIT intervention, trained study assistant will contact participants by phone at 6 and 12 months from date of enrollment for ascertainment of outcomes. The trained study assistant who interviews the participant will not be aware of the patient’s treatment assignment. Patients will be followed for a total of 12 months after SUMMIT introduction (up to 18 months if assignment to wait-list control group).

As a token of appreciation for the time spent completing the study survey, study participants will receive a $5 incentive for each survey administration completed. In addition, study participants who participate the focus group interview will receive a $10 incentive for their time. The incentive may be in the form of a debit card.

Source Records that will be used to collect data about participants:

This study will derive data from 6 sources:

1. Quantitative Survey instrument (see attachment A-B)- baseline, and follow-up survey to be administered at 6 and 12 months (attachment B). Stored electronically secure database (RedCap)
2. Existing Clinic Electronic Health Record (EHR). We will use existing EHR clinical data to track intervention process measures, clinical measurements (Hemoglobin A1C, blood pressure, phq-9, PAM, functional status/DLA20).
3. Oregon Health Plan (OHP) claims data. We will obtain claims data for all participants in the trial 6 months prior to consent and enrollment through 12 months follow-up after initiation into the SUMMIT intervention.
4. Death records. We will use death records to confirm the date and cause of death of participants who die during the trial period.
5. Quantitative Survey instrument for providers to be administered to providers at baseline,6-months and 12-months (attachment C). Stored electronically secure database (RedCap)
6. Qualitative semi-structured interview scripts of providers (see attachment D)
7. Qualitative semi-structure interview scripts of participants (see attachment E)

There are no anticipated circumstances under which subjects will be withdrawn from the research without their consent.

7) Data and Specimens

a) Handling of Data and Specimens

Data collection will be conducted using a laptop computer containing the survey instrument, or paper instrument as a backup (see attachment A-C, database template). Responses from paper instrument will then be programmed into a REDCap electronic database. This internet-based data management software will allow study coordinators to directly input data and response into the electronic database in a safe and secure manner with built in validation. Querying and reporting will be done by exporting de-identified data from the REDCap database into statistical software, R and STATA.

b) Sharing of Results with Subjects

At end of study, results can be shared with patient. During study, clinical information such as new diagnosis of depression, or functional status will be discussed with primary care teams (intervention or usual care) as well as patient as per usual clinical care guidelines.

c) Data and Specimen Banking

Data collected using the survey instrument will be destroyed at 6 years following the cut-off date (anticipated to be end of FY2018), according to Records Control Schedule policy. Medical records and claims data, which are part of day-to-day operations of OTC, will be stored according to regular protocol. No data will be stored for future unspecified uses.

8) Data Analysis

Targeted/Planned Enrollment
The expected target enrollment is 300 patient participants, and 80 provider/staff participants in the study.

Strategies to reduce error in intervention: SUMMIT team members are required to undergo training sessions prior to actual implementation of intervention. Study staff will also observe intervention team and meet with SUMMIT team periodically to assess changes to procedures over time. The wait-list control design will allow for standardized measurement of care received by those participating in the intervention compared to those participants in usual care prior to initiating the intervention. Control of the enrollment process and randomization of those assigned to waitlist or intervention group will help reduce selection bias and allow comparison between usual care and intervention participants, and allow for adjustment over time.

Unintentional unblinding of the primary outcomes ascertainment will be minimized by having study assistant adhere to the randomization protocol during baseline interview.

Outcome variables, blindly ascertained:

1. The primary aim from the health system perspective is to reduce inpatient hospitalization utilization and costs using a ‘Difference in Differences’ outcome
    a. Total inpatient costs over study period
    b. Total inpatient hospitalizations over study period
    c. Total number of patients who experience 30-day readmission during study period
    d. Total number of ambulatory sensitive condition admissions over study period

e. Average length of stay during inpatient hospitalization

2. PAM measures at follow up telephone survey done 6, and 12 months. The PAM scores and confidence intervals between intervention and control groups will be compared.
3. SF-12 quality of life measure
4. Patient experience as measured by the ambulatory consumer assessment of healthcare providers and systems (CAHPS)

Statistical Analysis Plan:
We will use chi-square tests to conduct univariate and bivariate analyses for categorical variables, and t-tests to compare continuous variables between the intervention and wait-list control groups. For our outcomes, we plan to use mean difference within difference comparisons between intervention and wait-list control groups. We will also use generalized estimating equation regression model to control for confounding variables of time entered into the intervention arm, length of follow-up time, patient characteristics, and clinical characteristics.

Sample Size Calculations

1) Annual inpatient hospitalization costs
    1. Null hypothesis: mean inpatient hospitalization costs after 12 months enrollment in the intervention will be the same as those treated in usual care
    2. Alternative hypothesis: mean inpatient hospitalization costs after 12 months enrollment in the intervention will be reduced by 10% compared to those who remain in the usual care
    3. Effect size = 4,200 (10% of 42,179)
    4. Standard deviation reported in VA study[18] (58,679)
    5. Standard effect size = 4200/ 60,000 = .07
    6. 2 sided alpha = 0.05, B = 1- .80 = .20
    7. sample size 1571

2) Patient Activation Measure (PAM)
    1. Null Hypothesis: An Ambulatory ICU intervention does not improve PAM scores by 4 points at 12 months
    2. Alt Hypothesis: An Ambulatory ICU intervention does not improve PAM scores by 4 pts at 3 months
    3. Effect size: 4 was measured in initial paper on PAM measure
    4. Standard deviation: 13 has been documented in prior studies using the PAM measurement[19]
    5. Alpha: 2 tailed 0.05; Beta: 0.20
    6. Sample size calculation: 196 patients (note if we change effect size to 3, sample size increases to 348)

3) SF-12
    1. Null: intervention will not increase SF-12 scores by 7 points
    2. Alt: intervention will increase SF-12 scores of participants at 12 months by 7 points (clinically significant)
    3. Effect size: 7 (0-100)
    4. SD: 25[20]
    5. Alpha: 2 tailed .05; Beta: 0.20 for power of 80
    6. Sample size calculation: 400 needed (306 needed to detect 8 point change)

 Template Revised: 6.15.2015

Quality control, data management, and administrative issues:

Pretest plans:

Prior to the SUMMIT intervention study, we will conduct a small pilot study of the intervention. In this pretest plan we will carry out the following:

1. Test the randomization protocol. If the patient meets criteria, then the study staff will approach the patient and begin the baseline interview. We will test the enrollment strategy until we have 10 patients in each group (wait list vs intervention)
2. Test and develop the baseline interview instrument. We will test the baseline instrument during this pilot study. Concerns arising from development of the instrument include the number of questions asked and concern for bias due to interviewer burnout, deviation from the script, ability to complete baseline interviews in setting of acute care.
3. Test and develop the follow up instrument. Because the main outcome is Patient Activation Measure (PAM) improvement after 6 and 12 months we will conduct the follow up interview by phone at 2 months. Although prior studies have shown utility of phone follow up in terms of patient interest in participation, we will need to see how much response rate is achieved. Prior studies have shown that patients do participate in phone follow up.[21] The pilot will also allow us to determine which questions will be asked at 6 months and 12 months, which may differ based on the pilot.
4. For both 2 and 3 above, staff administering baseline instrument and follow up instrument will be observed to see if the protocol directions are being followed and to observe any problems with conducting interviews.

Data Management:

Our data collection tool is developed first on paper (see attachment for example). Patient measurements were assessed using existing validated measures whenever possible (for example SF-12 for quality of life, Charlson scores for co-morbidity). The paper instrument will then be programmed into a REDCAP electronic database. This internet based data management software will allow study coordinators to directly input data and response into electronic database in a safe and secure manner with built in validation. Querying and reporting will be done by exporting data from REDCAP database into STATA.

Personnel/timetable:

It is anticipated that the study will require 1.5 FTE for study research assistants who will carry out recruitment, obtain consent, and baseline and follow up survey. The study will use existing OTC staff for intervention.

Institutional Background:

Oregon Health & Science University is a nonprofit public corporation.

The Old Town Clinic is an integrated medical and behavioral healthcare clinic for homeless individuals or those with very low incomes. OTC is one of 30 primary care practices recently selected as an

"exemplar practice" by the Robert Wood Johnson Foundation's LEAP Project. Over 3,500 individuals received services last year.

9) Privacy, Confidentiality and Data Security

Concerns of privacy and data security will be addressed by use of secure REDCap data management software, which is web-based software with security measures in place. All records that contain biographical information (i.e. address, phone lists, etc.) will be stored in this secure database. No identifying data will be included in publications or presentations. Access to all participant data and information will be restricted to authorized personnel only. Medical records at OTC are kept in a computer database that is password protected.

We may release this information to others outside of OHSU who are involved in conducting or overseeing research, including: The Office for Human Research Protections, a federal agency that oversees research involving humans.

Because we are collecting sensitive information from study subjects, which will be connected to identifying information, we will apply for a certificate of confidentiality from NIH. The certificate of confidentiality will allow us to resist the disclosure of sensitive or identifying information in any federal, state, or local civil, criminal, administrative, legislative, or other proceedings.

Data will be handled according to OHSU policies for data repositories. Each time data is released for research purposes a tracking sheet and sharing agreement will be used. Study data will be kept for 6 years after study completion to enable completion of analysis and write-up of results, after which this data will be destroyed.

10) VAPORHCS participation

VA employees, Samuel Edwards and Elizabeth Hulen, will be involved with participant interviews and data analysis for this project as co-investigator, and research assistant respectively. VA staff will be involved in the consent of participants to the study, administration of baseline and follow up surveys, and facilitation of qualitative interviews and data collection off VA time. The research activities that will occur at the VA Portland Health Care System (VAPORHCS) on VA time involve the management and analysis of quantitative and qualitative data. Quantitative data will be housed in a secure web-based REDCap database as described previously. Qualitative data will be housed at the VAPORHCS on a secure network drive assigned to Samuel Edwards. These qualitative data include field notes and semi-structured interviews previously transcribed into electronic documents. Data will be stripped of personal identifiers prior to their transcription and housing at the VAPORHCS. The VA resources that will be used for this study are employee work time, VA computers, office space (Bldg 6 Rm 326) and ATLAS.ti software. No VA patients will be enrolled, screened, or recruited for this study.

11) Risks and Benefits

a) Risks to Subjects

This study poses no more than minimal risk to subjects, as the probability and magnitude of harm or discomfort anticipated in the research are not greater than those encountered in routine care. Subjects may experience inconvenience from semi-annual surveys conducted in-person or over the phone. There also a risk of the breach of confidentiality.

b) Potential Benefits to Subjects

Study subjects may be able to improve their quality of life, health time management after discussion with the researchers. Subjects who screen positive for the survey baseline instruments including depression, cognition, substance use, housing insecurity, food insecurity will be flagged by the researchers and health team notified whether in the intervention or awaiting the intervention. The results from this study will guide efforts to improve the quality of care delivered at OTC.

Data Safety and Monitoring Plan

The questionnaire and intervention is noninvasive and not expected to cause any harm or side effects. The research team will produce administrative reports on a monthly basis that describe study progress including: accrual, demographic, study subjects status, outstanding study forms, error rate pertaining to adherence to inclusion/exclusion criteria and the study protocol. These reports will be reviewed internally for ongoing quality control. All hard-copy records for study participants such as informed consents will be kept in a locked office file cabinet in a secure office space at OTC.

Should the protocol be amended as a result of data review, the IRB will be notified and the amendment approved prior to study amendment implementation unless the protocol amendment must be implemented to protect the immediate safety of the study subjects.

ClinicalTrials.gov Requirements

The PI will be responsible for registration of this trial at ClinicalTrials.gov at such a time as IRB approval has been approved, and prior to enrolling patients for this study.

References

1. Cohen s, Yu w. The Concentration and Persistence in the Level of Health Expenditures over Time: Estimates for the U.S. Population, 2008–2009. Statistical Brief #354 Agency for Healthcare Research and Quality, Rockville, MD 2012.
2. Vogeli C, Shields AE, Lee TA, et al. Multiple chronic conditions: prevalence, health consequences, and implications for quality, care management, and costs. J Gen Intern Med 2007;22 Suppl 3:391-5.
3. Wensing M, Vingerhoets E, Grol R. Functional status, health problems, age and comorbidity in primary care patients. Quality of life research : an international journal of quality of life aspects of treatment, care and rehabilitation 2001;10:141-8.
4. Forster AJ, Murff HJ, Peterson JF, Gandhi TK, Bates DW. The incidence and severity of adverse events affecting patients after discharge from the hospital. Ann Intern Med 2003;138:161-7.

5. Freund T, Wensing M, Mahler C, et al. Development of a primary care-based complex care management intervention for chronically ill patients at high risk for hospitalization: a study protocol. Implementation science : IS 2010;5:70.

6. Jack BW, Chetty VK, Anthony D, et al. A reengineered hospital discharge program to decrease rehospitalization: a randomized trial. Ann Intern Med 2009;150:178-87.

7. Coleman EA, Parry C, Chalmers S, Min SJ. The care transitions intervention: results of a randomized controlled trial. Archives of internal medicine 2006;166:1822-8.

8. Naylor MD, Brooten D, Campbell R, et al. Comprehensive discharge planning and home follow-up of hospitalized elders: a randomized clinical trial. JAMA : the journal of the American Medical Association 1999;281:613-20.

9. Kangovi S, Barg FK, Carter T, Long JA, Shannon R, Grande D. Understanding why patients of low socioeconomic status prefer hospitals over ambulatory care. Health affairs 2013;32:1196-203.

10. Hong CS, Abrams MK, Ferris TG. Toward increased adoption of complex care management. The New England journal of medicine 2014;371:491-3.

11. Hong CS, Siegel AL, Ferris TG. Caring for high-need, high-cost patients: what makes for a successful care management program? Issue brief 2014;19:1-19.

12. Williams JW, Jackson GL, Powers BJ, et al. Closing the quality gap: revisiting the state of the science (vol. 2: the patient-centered medical home). Evidence report/technology assessment 2012:1-210.

13. Yee T, Lechner, AE., Carrier E. High-intensity Primary Care: Lessons for Physician and Patient Engagement. (National Institute for Health Care Reform Research Brief No 9) Retrieved from http://wwwnihcrorg/High-Intensity-Primary-Care Accessed Nov 12, 2015 2012.

14. Peterson K HM, HUmphrey L, Christensen V, Carson S. . Evidence Brief: Effectiveness of Intensive Primary Care Programs, VA-ESP Project #09-199. 2013.

15. Saitz R, Cheng DM, Winter M, et al. Chronic care management for dependence on alcohol and other drugs: the AHEAD randomized trial. JAMA : the journal of the American Medical Association 2013;310:1156-67.

16. Brown CA, Lilford RJ. The stepped wedge trial design: a systematic review. BMC medical research methodology 2006;6:54.

17. Handley MA, Schillinger D, Shiboski S. Quasi-experimental designs in practice-based research settings: design and implementation considerations. Journal of the American Board of Family Medicine : JABFM 2011;24:589-96.

18. Zulman DM, Pal Chee C, Wagner TH, et al. Multimorbidity and healthcare utilisation among high-cost patients in the US Veterans Affairs Health Care System. BMJ open 2015;5:e007771.

19. Maindal HT, Sandbaek A, Kirkevold M, Lauritzen T. Effect on motivation, perceived competence, and activation after participation in the "Ready to Act" programme for people with screen-detected dysglycaemia: a 1-year randomised controlled trial, Addition-DK. Scandinavian journal of public health 2011;39:262-71.

20. Bergmo TS, Berntsen GK, Dalbakk M, Rumpsfeld M. The effectiveness and cost effectiveness of the PAtient-Centred Team (PACT) model: study protocol of a prospective matched control before-and-after study. BMC geriatrics 2015;15:133.

21. Flacker J, Park W, Sims A. Hospital discharge information and older patients: do they get what they need? Journal of hospital medicine : an official publication of the Society of Hospital Medicine 2007;2:291-6.
