# Supplementary material for: Ambulatory Intensive Care for Medically Complex Patients at a Health Care Clinic for Individuals Experiencing Homelessness: The SUMMIT Randomized Clinical Trial
Source: JAMA Netw Open. 2023 Nov 10;6(11):e2342012. doi: 10.1001/jamanetworkopen.2023.42012 (PMC10638646; doi:10.1001/jamanetworkopen.2023.42012)
Supplement: Supplement 2. — eAppendix 1. Core Activities of the Streamlined Unified Meaningfully Managed Interdisciplinary Team (SUMMIT) Ambulatory Intensive Care Unit (A-ICU) eAppendix 2. Study Enrollment Criteria eTable 1. Outcomes and Assessment Instruments eTable 2. Outlier Sensitivity Analysis for Administrative and Patient-Reported Outcomes at 6 Months From a Linear Mixed-Effects Model eTable 3. Missing Data Sensitivity Analysis of Baseline Characteristics of Those Missing Patient-Reported Outcomes at 6 Months eReferences [file jamanetwopen-e2342012-s002.pdf]

Supplementary Online Content

Chan B, Edwards ST, Srikanth P, et al. Ambulatory intensive care for medically complex patients at a health care clinic for individuals experiencing homelessness: the SUMMIT randomized clinical trial. *JAMA Netw Open*. 2023;6(11):e2342012. doi:10.1001/jamanetworkopen.2023.42012

eAppendix 1. Core Activities of the Streamlined Unified Meaningfully Managed Interdisciplinary Team (SUMMIT) Ambulatory Intensive Care Unit (A-ICU)
eAppendix 2. Study Enrollment Criteria
eTable 1. Outcomes and Assessment Instruments
eTable 2. Outlier Sensitivity Analysis for Administrative and Patient-Reported Outcomes at 6 Months From a Linear Mixed-Effects Model
eTable 3. Missing Data Sensitivity Analysis of Baseline Characteristics of Those Missing Patient-Reported Outcomes at 6 Months
eReferences

This supplementary material has been provided by the authors to give readers additional information about their work.

© 2023 Chan B et al. *JAMA Network Open*.

eAppendix 1. Core Activities of the Streamlined Unified Meaningfully Managed Interdisciplinary Team (SUMMIT) Ambulatory Intensive Care Unit (A-ICU)

1. **Transfer of care to the co-located stand-alone team.** Patients transfer care from existing primary care to the SUMMIT team to encourage coordinated, unified care from a single team. Co-location offers opportunity to facilitate interdisciplinary meetings, as well as informal conversations to enact care plans during non-visit time.
2. **Comprehensive initial intake.** The first visit(s) include a 60 minute social intake with the Social worker, followed by a 60 minute medical intake with provider/care coordinator with open ended questions and focus on patient health goal elicitation and assessing self-efficacy and treatment burden. The visit forms the basis for the patient-centered, goal-based care plan
3. **Interdisciplinary team reviews.** Following intake, subsequent activities and appointments are determined based on patient status, medical and psychosocial complexity. A 15 minute scheduled daily huddle provide opportunity to discuss the patients scheduled for the day, and recently hospitalized or discharged patients. There is also a weekly 2-hour interdisciplinary rounds that provide opportunity to review existing patients to assess whether current interventions are working and revise care plans as necessary.
4. **Transitions of care coordination/tracking**. Led by the complex care nurse, and pharmacist, the SUMMIT team developed protocols for coordination of care for hospitalized patients to communicate pertinent information to inpatient care teams, and develop follow up care plans prior to patient discharge.
5. **Built-in counseling services**. Led by the social worker but supported by all team members who are trained in motivational interviewing, trauma-informed care, and cognitive behavioral therapy. Social workers provide individualized counseling and leverage existing linkages to mental health prescribers as necessary.
6. **Navigation of social services**. Team members assist patients with long term care planning, advanced directives, linkages to community resources such as disability, housing benefits.
7. **On-demand Availability and access to off-hours warm lines**. A separate SUMMIT team phone number is available for patients and patient caregiver teams to access SUMMIT team members at all hours of the day. SUMMIT physicians cover the phone during off-clinic hours to respond to patient care needs and concerns.

Additional Activities / Flexibility

8. **Outreach visits.** Team members are available to conduct outreach visits for patients on an as needed basis. Visits are used as an opportunity to assess patients outside the clinic, develop rapport and trust, and facilitate and support a health related activity (i.e., Accompaniment to specialist referrals, delivery of medications if temporarily homebound, assistance with access to social services).
9. **Pharmacy education/ chronic disease medication management**. For select patients with non-controlled chronic disease conditions (diabetes, hypertension, heart disease), the SUMMIT pharmacist is available for 1 to 1 consultation and medication review and management. The pharmacist is empowered to enact the care plan and titration or tapering of medications.
10. **In-visit scribing**. Care coordinators sit in on medical visits with provider and patients and scribe for the provider. This activity promotes unified communication of the care plan between patient, care coordinator, and physician; and allows improved patient experience during face-to-face visits.

© 2023 Chan B et al. *JAMA Network Open*.

eAppendix 2. Study Enrollment Criteria

Enrollment Criteria:

- 1 or more medical/surgical hospitalizations in last 6 months
- 1 or more of the following medical conditions:
    - Chronic Kidney Disease stage III
    - Congestive Heart Failure
    - Chronic Obstructive Pulmonary Disease, group C/D
    - Diabetes with A1c > 8%
    - End Stage Liver Disease (ESLD)
    - Osteomyelitis/severe soft-tissue infection
- Or 1 medical condition and one or more of the following co-morbid conditions
    - Mental health diagnosis
    - Substance use disorder diagnosis

Exclusion Criteria:

- Inability to consent (as demonstrated by "teach-back" method)
- Non-English speaking patients
- On hospice or deemed < 6 months to live at time of consent/enrollment
- Diagnosis of terminal cancer
- Inability to participate in follow up assessment due to aphasia, severe hearing impairment or behavioral issues.

© 2023 Chan B et al. *JAMA Network Open*.

eTable 1. Outcomes and Assessment Instruments

Outcome:	Definition/Instrument:	Calculation:	Notes/Comment:
**Primary Outcome**			
Hospitalization rate	Change in hospitalization rates at 6-months vs baseline	# of hospitalizations/6-months post enrollment - # hospitalizations/6-months prior to enrollment	Patients who died prior to 6-month follow-up had rate calculated by # of hospitalizations/months contributed<br><br>Data obtained from regional health information exchange (HIE) that fed into clinic administrative data
**Secondary Utilization Outcomes**			
Emergency Visit rate (ED)	Change in ED visit rates at 6-months vs baseline	# of ED visits/6-months post enrollment - # ED visits/6-months prior to enrollment	Patients who died prior to 6-month follow-up had rate calculated by # of ED visits/months contributed<br><br>Data obtained from clinical data warehouse
Primary Care Visit rates (PCP visits)	Change in PCP visit rates at 6-months vs baseline	# of PCP visits/6-months post enrollment - # PCP visits/6-months prior to enrollment	Patients who died prior to 6-month follow-up had rate calculated by # of PCP visits/months contributed<br><br>Data obtained from clinical data warehouse
Behavioral Health Visit rates (BH visits)	Change in BH visit rates at 6-months vs baseline	# of BH visits/6-months post enrollment - # BH visits/6-months prior to enrollment	Patients who died prior to 6-month follow-up had rate calculated by # of BH visits/months contributed<br><br>Data obtained from clinical data warehouse
**Secondary Patient Reported Outcomes**			
Patient Activation	Patient Activation Measure (PAM-10) [1,2]<br><br>Change at 6-months vs baseline	10 item questionnaire with Likert scale response from 1 (strongly disagree) to 4 (strongly agree). Raw score is divided by # of questions answered and multiplied by 10 to obtain a 1-100 score; higher scores indicate higher patient activation	Assessed at baseline and at 6-month via survey<br><br>An improvement in 4 points is considered a minimal clinically important difference
Patient Experience	Ambulatory Consumer Assessment of Healthcare Providers and Systems (A-CAHPS) [2]	10 item questionnaire encompassing 3 domains (provider/team communication, access, and coordination) with	Assessed at baseline and at 6-month via survey

© 2023 Chan B et al. *JAMA Network Open*.

	Change at 6-months vs baseline	Likert scale response from 1 (never) to 4 (always), linear mean scoring (0-100) where higher score indicates higher patient experience	
Patent Reported Quality of Life	12-item short form health survey (SF-12) [3]<br><br>Change at 6-months vs baseline	12 questions that encompass 2 summary composite scores (physical and mental health) with 8 sub-domain (e.g. bodily pain, role emotional, social functioning. Composite scores are represented as a T-score with mean of 50 and standard deviation of 10	Assessed at baseline and at 6-month via survey
Palliative Care Self-rated Health	One question item from the Edmonton Symptom Assessment System (ESAS) [4]<br><br>Change at 6-months vs baseline	"Please choose the number that best describes how you feel now, (0 being worst possible wellbeing, 10 being best wellbeing)"	Assessed at baseline and at 6-month
**Selected Baseline Assessments and Psychosocial Measures**			
Social Support	ENRICHD Social Support Instrument (ESSI) [5]	7-item survey with Likert scale scoring (1-5) for 6-items and yes/no for item 7. Scores were linear transformed to create a score from 0-100, higher indicates higher social support	Assessed at baseline
Cognitive Impairment	Telephone interview for cognitive status (TICS) [6]	11-item test that is summed with score ranges from 0-41; we defined cognitive impairment as a score < 20	Assessed at baseline
Health literacy	Brief health literacy screening tool by Chew[7,8]	3-item survey with Likert scale scoring. We defined inadequate health literacy as a response of "not at all/a little bit/moderately" to "Confidence with Forms" question	Assessed at baseline
Food insecurity	2-item Food Insecurity (FI) screening tool by Hagar et al. [9]	2-item survey with yes/no response; We defined food insecurity	Assessed at baseline

© 2023 Chan B et al. *JAMA Network Open*.

		as a positive screen to either question	

© 2023 Chan B et al. *JAMA Network Open*.

eTable 2. Outlier Sensitivity Analysis for Administrative and Patient-Reported Outcomes at 6 Months From a Linear Mixed-Effects Model[a]

	Immediate			EUC				
	Baseline	6-month	Within-group change	Baseline	6-month	Within-group change	Difference (95% CI)[b]	P[b]
N	77			75				
Hospitalization rate	2.3 (0.2)	1.9 (0.2)	-0.4 (0.3)	1.9 (0.2)	1.6 (0.2)	-0.3 (0.3)	-0.1 (-0.8, 0.7)	0.89
N	72			78				
ED visit rate	3.57 (0.5)	3.3 (0.5)	-0.3 (0.6)	3.5 (0.5)	2.9 (0.5)	-0.6 (0.6)	0.3 (-1.3, 1.9)	0.73
N	65			77				
Primary care visit	5.2 (0.7)	9.6 (0.7)	4.5 (0.7)[d]	6.2 (0.7)	5.5 (0.7)	-0.7 (0.7)	5.2 (3.2, 7.2)	<0.001

[a]random intercept for subject; [b]between SUMMIT and EUC; [c]p<0.05; [d]p<0.01

© 2023 Chan B et al. *JAMA Network Open*.

eTable 3. Missing Data Sensitivity Analysis of Baseline Characteristics of Those Missing Patient-Reported Outcomes at 6 Months

	**Total N=159**	**Non-Missing N=123**	**Missing N=36**
Sex[a], n (%)			
Male	102 (65.8%)	74 (62.2%)	28 (77.8%)
Female	53 (34.2%)	45 (37.8%)	8 (22.2%)
Age (years), mean (SD)	54.9 (9.8)	55.4 (9.8)	53.2 (9.6)
Social Support (ENRICHD)[a], mean (SD)	18.9 (6.7)	19.0 (6.7)	18.2 (6.8)
Gross HH Income ≤$1,000, n (%)	133 (85.3%)	102 (83.6%)	31 (91.2%)
High School Educ or Less, n (%)	96 (61.1%)	74 (60.7%)	22 (62.9%)
Self-rated Health, mean (SD)	5.3 (2.3)	5.4 (2.3)	4.7 (2.3)
SF12:Aggregate Physical Health Composite[a], mean (SD)	27.2 (9.2)	26.8 (8.7)	28.7 (10.8)
SF12:Aggregate Mental Health Composite[a], mean (SD)	40.1 (13.0)	39.7 (12.7)	41.2 (14.2)
Patient Activation Measure[a], mean (SD)	55.6 (12.0)	55.1 (11.7)	57.3 (12.8)
Cognitive Impairment[a,e], n (%)	63 (40.6%)	49 (40.2%)	14 (42.4%)
Race/Ethnicity[b], n (%)			
Asian	3 (1.9%)	3 (2.4%)	0 (0.0%)
Black/African American	20 (12.6%)	15 (12.2%)	5 (13.9%)
Hispanic/Latino	5 (3.1%)	5 (4.1%)	0 (0.0%)
Native American/Native Alaskan	20 (12.6%)	15 (12.2%)	5 (13.9%)
Native Hawaiian/Pacific Islander	1 (0.6%)	1 (0.8%)	0 (0.0%)
White	121 (76.1%)	94 (76.4%)	27 (75.0%)
Other	5 (3.1%)	4 (3.3%)	1 (2.8%)
Drug Abuse Screening[a,c], n (%)			
No problems reported	62 (39.5%)	50 (41.0%)	12 (34.3%)
Low/Moderate Level	56 (35.7%)	41 (33.6%)	15 (42.9%)
Substantial/Severe Level	39 (24.8%)	31 (25.4%)	8 (22.9%)
Current Alcohol Problem[a,c], n (%)	32 (20.5%)	24 (19.8%)	8 (22.9%)
Current Residence[a], n (%)			
Sleeping Outside/Place not meant for habitation	21 (13.4%)	18 (14.9%)	3 (8.6%)
Shelter	15 (9.6%)	13 (10.7%)	2 (5.7%)
Transitional housing	28 (18.0%)	16 (13.2%)	12 (34.3%)
Permanent housing	62 (39.8%)	45 (45.4%)	7 (20.0%)
With friends/family	8 (5.1%)	5 (4.1%)	3 (8.6%)
Nursing facility/assisted living	22 (14.1%)	14 (11.6%)	8 (22.9%)
Housing Stability[a,g], n (%)			
Stable housing	92 (59.0%)	64 (52.0%)	18 (54.5%)
Unstable housing	64 (41.0%)	59 (48.0%)	15 (45.5%)
Depression, n (%)	84 (53.8%)	67 (55.8%)	17 (47.2%)
Elixhauser Comorbidity Score, n (%)			
<0	59 (38.1%)	48 (40.0%)	11 (31.4%)
0	4 (2.6%)	3 (2.5%)	1 (2.9%)
1-4	38 (24.5%)	29 (24.2%)	9 (25.7%)
>=5	54 (34.8%)	40 (33.3%)	14 (40.0%)

[f]EUC- enhanced usual care

© 2023 Chan B et al. *JAMA Network Open*.

[a]There were 4 unknown/decline sex, 2 missing social support, 3 missing/decline HH income, 2 missing education, 1 missing self-rated health, 7 missing SF12 aggregate physical and mental health, 1 missing patient activation measure, 4 missing cognitive impairment, 2 missing drug abuse screening, 3 missing alcohol problem, 3 missing/decline current residence, 3 missing depression, 4 missing Elixhauser Comorbidity Score,

[b]Percentages may not add up to 100 because of rounding and multiple race/ethnicity categories checked.

[c]Drug abuse screening test (DAST-10) for drug abuse screening problem & alcohol use disorders identification test (AUDIT-10) for alcohol problems.

[d]Household Income in the past month

[e] Defined as Telephone Interview Cognitive Status (TICS) score of <20

[g]Unstable Housing defined as Sleeping Outside/Place not meant for habitation,Shelter, Transitional Housing; Stable Housing defined as permanent housing, Staying with friends or family, Nursing facility/assisted living.

© 2023 Chan B et al. *JAMA Network Open*.

eReferences

1. Hibbard JH, Mahoney ER, Stockard J, Tusler M. Development and testing of a short form of the patient activation measure. *Health services research*. Dec 2005;40(6 Pt 1):1918-30. doi:10.1111/j.1475-6773.2005.00438.x
2. Giordano LA, Elliott MN, Goldstein E, Lehrman WG, Spencer PA. Development, implementation, and public reporting of the HCAHPS survey. *Medical care research and review : MCRR*. Feb 2010;67(1):27-37. doi:10.1177/1077558709341065
3. Ware J, Jr., Kosinski M, Keller SD. A 12-Item Short-Form Health Survey: construction of scales and preliminary tests of reliability and validity. *Medical care*. Mar 1996;34(3):220-33.
4. Bruera E, Kuehn N, Miller MJ, Selmser P, Macmillan K. The Edmonton Symptom Assessment System (ESAS): a simple method for the assessment of palliative care patients. *Journal of palliative care*. Summer 1991;7(2):6-9.
5. Berkman LF, Blumenthal J, Burg M, et al. Effects of treating depression and low perceived social support on clinical events after myocardial infarction: the Enhancing Recovery in Coronary Heart Disease Patients (ENRICHD) Randomized Trial. *JAMA : the journal of the American Medical Association*. Jun 18 2003;289(23):3106-16. doi:10.1001/jama.289.23.3106
6. Roccaforte WH, Burke WJ, Bayer BL, Wengel SP. Validation of a telephone version of the mini-mental state examination. *Journal of the American Geriatrics Society*. Jul 1992;40(7):697-702.
7. Chew LD, Bradley KA, Boyko EJ. Brief questions to identify patients with inadequate health literacy. *Fam Med*. Sep 2004;36(8):588-94.
8. Chew LD, Griffin JM, Partin MR, et al. Validation of screening questions for limited health literacy in a large VA outpatient population. *J Gen Intern Med*. May 2008;23(5):561-6. doi:10.1007/s11606-008-0520-5
9. Hager ER, Quigg AM, Black MM, et al. Development and validity of a 2-item screen to identify families at risk for food insecurity. *Pediatrics*. Jul 2010;126(1):e26-32. doi:10.1542/peds.2009-3146

© 2023 Chan B et al. *JAMA Network Open*.
