# Supplementary material for: Ambulatory Intensive Care for Medically Complex Patients at a Health Care Clinic for Individuals Experiencing Homelessness: The SUMMIT Randomized Clinical Trial
Source: JAMA Netw Open. 2023 Nov 10;6(11):e2342012. doi: 10.1001/jamanetworkopen.2023.42012 (PMC10638646; doi:10.1001/jamanetworkopen.2023.42012)
Supplement: Supplement 3. — Data Sharing Statement [file jamanetwopen-e2342012-s003.pdf]

Data Sharing Statement

Chan. Ambulatory Intensive Care for Complex Patients at a Health Care Clinic for Individuals Experiencing Homelessness. *JAMA Netw Open*. Published November 10, 2023. doi:10.1001/jamanetworkopen.2023.42012

Data
Data available: Yes
Data types: All deidentified participant data collected that underlie results reported in this article
How to access data: Requests for a deidentified dataset with proposal for how the data would be used/analysis plan can be sent to chanbri@ohsu.edu for consideration.
When available: Immediately following publication, no end date, upon request

Supporting Documents
Document types: Study protocol, informed consent forms, and analytic code

Additional Information
Who can access the data: Researchers who present a methodologically sound proposal researchers whose proposed use of the data has been approved
Types of analyses: To achieve aims of the approved proposal
Mechanisms of data availability: Proposals should be directed to chanbri@ohsu.edu, data requestors may need to sign a data use agreement. Data are available for 5 years and would be sent to the requester via email
